# Design of a Duplex-to-Complex Structure-Switching Approach for the Homogeneous Determination of Marine Biotoxins in Water

**DOI:** 10.3390/toxins16110476

**Published:** 2024-11-04

**Authors:** Awatef Al-Tabban, Amina Rhouati, Amjad Fataftah, Dana Cialla-May, Jürgen Popp, Mohammed Zourob

**Affiliations:** 1 Department of Chemistry, Alfaisal University, Al Zahrawi Street, Al Maather, Al Takhassusi Rd, Riyadh 11355, Saudi Arabia; aaltabban@alfaisal.edu (A.A.-T.); arhouati@alfaisal.edu (A.R.); afataftah@alfaisal.edu (A.F.); 2 Institute of Physical Chemistry (IPC) and Abbe Center of Photonics (ACP), Friedrich Schiller University Jena, Member of the Leibniz Centre for Photonics in Infection Research (LPI), Helmholtzweg 4, 07743 Jena, Germany; dana.cialla-may@uni-jena.de (D.C.-M.); juergen.popp@uni-jena.de (J.P.); 3 Bioengineering Laboratory, Higher National School of Biotechnology, Constantine 25100, Algeria; 4 Leibniz Institute of Photonic Technology, Member of Leibniz Health Technologies, Member of the Leibniz Centre for Photonics in Infection Research (LPI), Albert-Einstein-Straße 9, 07745 Jena, Germany

**Keywords:** marine toxins, aptamers, fluorescence, quenching, water

## Abstract

In this report, we describe a fluorescent assay for the detection of six marine toxins in water. The mechanism of detection is based on a duplex-to-complex structure-switching approach. The six aptamers specific to the targeted cyanotoxins were conjugated to a fluorescent dye, carboxyfluorescein (FAM). In parallel, complementary DNA (cDNA) sequences specific to each aptamer were conjugated to a fluorescence quencher BHQ1. In the absence of the target, an aptamer–cDNA duplex structure is formed, and the fluorescence is quenched. By adding the toxin, the aptamer tends to bind to its target and releases the cDNA. The fluorescence intensity is consequently restored after the formation of the complex aptamer–toxin, where the fluorescence recovery is directly correlated with the analyte concentration. Based on this principle, a highly sensitive detection of the six marine toxins was achieved, with the limits of detection of 0.15, 0.06, 0.075, 0.027, 0.041, and 0.026 nM for microcystin-LR, anatoxin-α, saxitoxin, cylindrospermopsin, okadaic acid, and brevetoxin, respectively. Moreover, each aptameric assay showed a very good selectivity towards the other five marine toxins. Finally, the developed technique was applied for the detection of the six toxins in spiked water samples with excellent recoveries.

## 1. Introduction

Cyanobacteria, also called blue-green algae, are photosynthetic microorganisms found in water bodies worldwide [1]. While they are essential to many aquatic ecosystems, some cyanobacteria species produce potent toxins, known as cyanotoxins, which threaten human and animal health [2]. Eutrophication, driven by nutrient pollution, particularly phosphorus and nitrogen, is a major factor contributing to cyanobacterial blooms [3]. These blooms can lead to hypoxia, loss of biodiversity, and the production of large quantities of toxins [4]. Cyanotoxins can be found in drinking water, recreational activities, and contaminated food, including fish and seafood [5]. Humans and animals exposed to these toxins can suffer from various forms of damage, including hepatotoxicity, nephrotoxicity, and neurotoxicity [6].

Cyanotoxins are classified according to their chemical structure and biological effects. The most well-studied cyanotoxins include microcystins: cyclic heptapeptides produced by various cyanobacterial species such as *Microcystis*, *Anabaena*, and *Planktothrix* [7]. Microcystins are potent inhibitors of protein phosphatases, leading to liver damage and promoting tumor growth even at low concentrations [8]. Microcystin-Leucine-Arginine (MC-LR) is the most toxic and commonly studied variant [9]. Cylindrospermopsins (CYN), also known for their hepatotoxicity, are commonly produced by *Cylindrospermopsis raciborskii* and *Aphanizomenon ovalisporum* [10]. In parallel, it has been demonstrated that CYN has cytotoxic and genotoxic effects [11]. Besides hepatotoxicity, some cyanotoxins classes have neurotoxic effects [12]. Anatoxins (ANTXs) act as potent agonists at nicotinic acetylcholine receptors, leading to paralysis and potentially fatal respiratory failure [13]. Anatoxins, including anatoxin-a, anatoxin-a (S), and homoanatoxin-a are produced by *Anabaena*, *Oscillatoria*, and *Aphanizomenon* species [14]. Known as paralytic shellfish compounds, saxitoxins (STXs) are also neurotoxic, able to block sodium channels in nerve cells [15]. Finally, brevetoxins (BTXs), liphophylic polyether toxins, have been also reported as neurotoxic cyanotoxins. They are produced by the marine dinoflagellate *Gymnodinium breve* [16,17]. In addition to cyanotoxins, okadaic acids (OAs) constitute a very important class of marine biotoxins worldwide. OAs are causative agents of diarrhetic seafood poisoning, produced by microalgae, *dinoflagellates Dinophysis* spp. and *Prorocentrum* spp. [18]. Okadaic acid was also considered a potential inhibitor of phosphatases 1 and 2A, causing the hyperphosphorylation of tau protein, leading to Alzheimer’s disease [19].

Detecting these toxins is crucial for ensuring the safety of drinking water, recreational water, and seafood. Various techniques have been developed to detect cyanobacteria toxins, each with its own strengths and limitations [20]. Toxin bioassays involve the use of living organisms, such as fish, mice, or cultured cells, to detect the presence of cyanotoxins based on their toxic effects [21,22]. While bioassays can provide information on the overall toxicity of a sample, they are not specific to individual toxins and raise ethical concerns due to the use of live animals [23]. Assays based on whole cells, which monitor the cytotoxic effects of toxins on cultured cells, offer a more ethical and specific alternative, though they still lack the precision of other methods such as immunoassays or chromatography and mass spectrometry techniques [24]. Immunochemical techniques, such as enzyme-linked immunosorbent assay (ELISA), are widely used for the detection of cyanotoxins. These assays are based on the specific binding of antibodies to cyanotoxins, which can be quantified to determine toxin concentrations. ELISA is relatively simple, cost-effective, and can be used for high-throughput screening [25]. However, immunoassays may sometimes lack specificity due to cross-reactivity with similar compounds, which can lead to false positives or negatives [26]. Chromatographic techniques such as HPLC (high-throughput liquid chromatography) and LC-MS (liquid chromatography coupled to mass spectrometry) are also used to separate and quantify marine toxins. LC-MS is a highly sensitive and specific technique used for detecting and quantifying multiple cyanotoxins simultaneously. Toxins are separated according to their chemical properties by liquid chromatography, then identified and quantified based on their mass-to-charge ratio using mass spectrometry [27]. Despite its sensitivity, LC-MS is also expensive, requires specialized equipment and expertise, and is not well suited for rapid field testing. Polymerase chain reaction (PCR) techniques have been used for detecting cyanobacteria by targeting their genomes or specific genes involved in toxin production, such as the *mcy* gene cluster responsible for microcystin biosynthesis [28,29]. PCR is valuable for early warning and monitoring purposes; however, it requires specialized equipment and technical expertise.

In addition to PCR-based detection methods, nucleic acids have attracted great interest in the field of biosensing. They can be used in DNA hybridization biosensors targeting a complementary DNA or RNA. In parallel, they can be used as bioreceptors called ‘aptamers’ in affinity-based biosensors to target different classes of molecules, cells, or microorganisms [30]. Aptamers are synthetic oligonucleotides generated by the SELEX process, and are characterized by their high affinity, specificity, and stability [31]. Their easy synthesis and outstanding binding properties make them a good alternative for antibodies in modern bioanalysis [32]. Various aptamers have been generated for a wide range of molecules, including marine toxins [33]. Given the chemical nature of aptamers, the latter can be labeled with different probes and chemical groups, allowing their use with electrochemical and optical detection methods [34]. Recently, we reported an electrochemical aptasensing platform for the simultaneous determination of five cyanotoxins in water [35]. It is worth noting that many cyanotoxins are usually present in the same sample, and multiplex detection is of great importance for ensuring water safety. Herein, we developed a fluorescent aptamer-based assay for the detection of six important marine toxins present in water (MC-LR, ANTX, SXT, CYN, OA, and BTX). The developed assay takes advantage of the capability of nucleic acids to form duplex structures based on the Watson–Crick helix. For this purpose, the aptamer sequences labeled with a fluorescent probe were used, and combined with their corresponding cDNA labeled with a fluorescent quencher. Following the FRET (fluorescence resonance energy transfer) principle, the duplex formation leads to fluorescence extinction. However, since aptamers’ affinity for their targets is much higher than their affinity for the short cDNA used, the complex fluorescent aptamer–toxin is formed, releasing the quencher-labeled cDNA and thus restoring the fluorescence intensity. Based on this fluorescent duplex-to-complex structure-switching approach, we achieved a highly sensitive detection of the six marine toxins within the wide linear range of 0.05 to 100 nM.

## 2. Results and Discussion

### 2.1. Principle of the Aptamer-Based Assay

**Figure 1:**
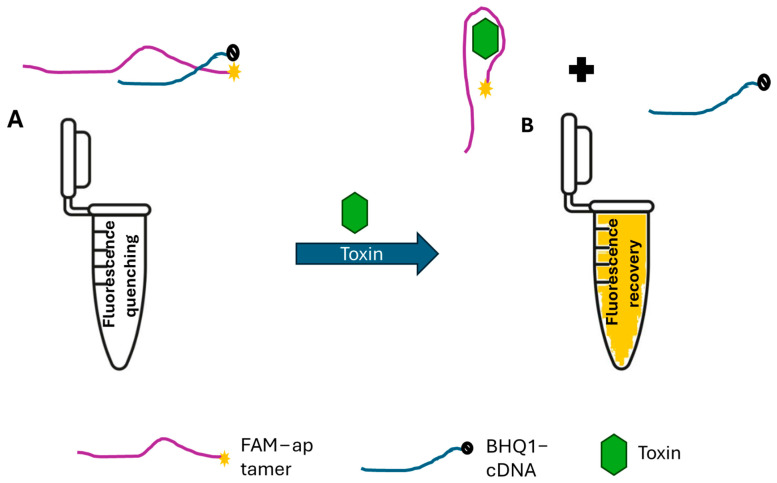
Sensing principle of the fluorescence aptameric assay. (**A**) Fluorescence quenching induced by the BHQ1-labeled cDNA sequence after the formation of the aptamer–cDNA duplex. (**B**) Fluorescence recovery after formation of the aptamer–toxin complex.

Figure 1 depicts the sensing mechanism of the fluorescent aptameric assay. The detection strategy is based on a duplex-to-complex structure-switching approach. Before the addition of the toxin, the fluorescent ssDNA aptamer binds to its complementary sequence to form a duplex structure. Therefore, BHQ1 conjugated to the 5′ end of the cDNA quenches the fluorescence of the aptamer by a transfer of energy phenomenon. In the presence of the target, the aptamer changes its conformation to form a complex with its target. Consequently, it releases the BHQ1-labeled cDNA, thus inducing the recovery of the FAM fluorescence. Therefore, the toxin amount can be measured by monitoring the increasing fluorescence intensity. Herein, we explore this sensing strategy for the detection of six cyanotoxins: microcystin-LR, anatoxin-α, saxitoxin, cylindrospermopsin, okadaic acid, and brevetoxin.

**Figure 2:**
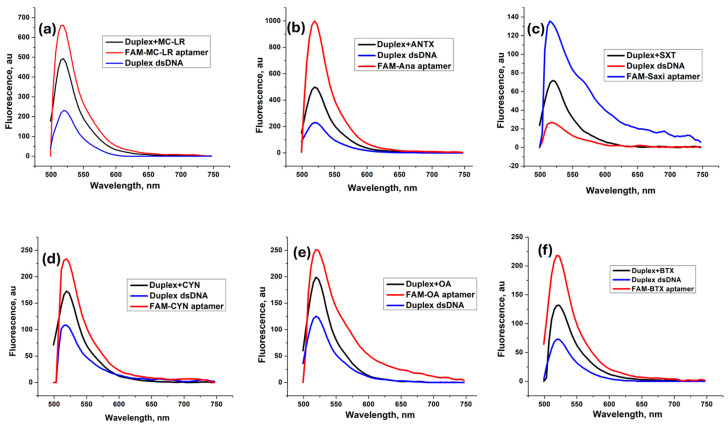
Fluorescence spectra of the FAM–aptamers, the aptamer–cDNA duplex, and the aptamer–target complex: microcystin-LR (**a**), anatoxin (**b**), saxitoxin (**c**), cylindrospermopsin (**d**), okadaic acid (**e**), and brevetoxin (**f**). Fluorescence measurements were performed at an emission wavelength of 518 nm.

To study the mechanism of detection, a proof-of-concept experiment was performed. Each FAM-labeled aptamer was subjected to a fluorescence measurement before and after formation of the duplex. Further, the fluorescence intensity was assessed after incubation of the duplex with a fixed concentration of the targeted toxin (5 nM). Figure 2a–f show the fluorescence spectra recorded at 518 nm, for the six toxins. The figures show a high fluorescence intensity in the red lines corresponding to the free FAM–aptamers. A significant drop in the fluorescence intensity was noted after the addition of the BHQ1-labeled cDNA (blue line). This decrease returns to the quenching efficiency of BHQ1 on the fluorophore and confirms the formation of the aptamer–cDNA duplex. Subsequently, the fluorescence was restored (black line), validating the binding between the aptamer and the target. The quenching efficiency was calculated for each duplex as ((F_0_ − F)/F_0_)%, where F_0_ is the fluorescence intensity of the aptamer and F is the fluorescence intensity of the aptamer–cDNA duplex. The quenching percentages were over 67%, except for CYN and OA, where the quenching efficiency was 54% and 50%, respectively.

### 2.2. Analytical Performance

**Figure 3:**
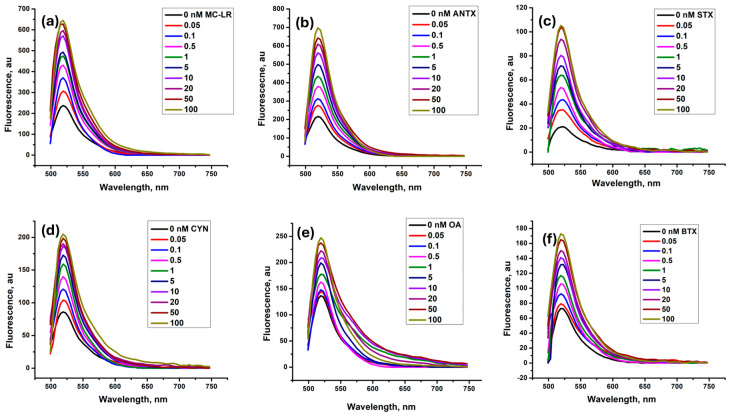
Fluorescence spectra of the six FAM–aptamers after incubation with increasing concentrations of their specific targets: microcystin-LR (**a**), anatoxin (**b**), saxitoxin (**c**), cylindrospermopsin (**d**), okadaic acid (**e**), and brevetoxin (**f**). Fluorescence measurements were performed at an emission wavelength of 518 nm.

Under the optimized conditions, the duplex-to-complex structure-switching approach was applied for the six toxins by designing the corresponding duplexes based on their aptamers and cDNA sequences labeled with FAM and BHQ1, respectively. For each toxin, the aptameric assay was performed in the absence and presence of different concentrations of the target. Figure 3a–f display the fluorescence spectra recorded for microcystin-LR, anatoxin-α, saxitoxin, cylindrospermopsin, okadaic acid, and brevetoxin, respectively. As expected, we observed that the fluorescence intensity increases by increasing the toxin concentration from 0.05 to 100 nM. For the six toxins, we noted a very low fluorescence intensity in the absence of the target. This is mainly due to the strong quenching effect of BHQ1 on the fluorophore resulting from the formation of the aptamer–cDNA duplex. By increasing the toxins’ concentrations, the fluorescence intensities increased gradually at the emission wavelength of 518 nm. This increase confirms the specific binding of the different toxins to their aptamers, leading to the dissociation of the duplex and the recovery of the FAM fluorescence. This returns to the fact that the aptamer’s affinity toward the target is much higher than its affinity for its cDNA sequence.

**Figure 4:**
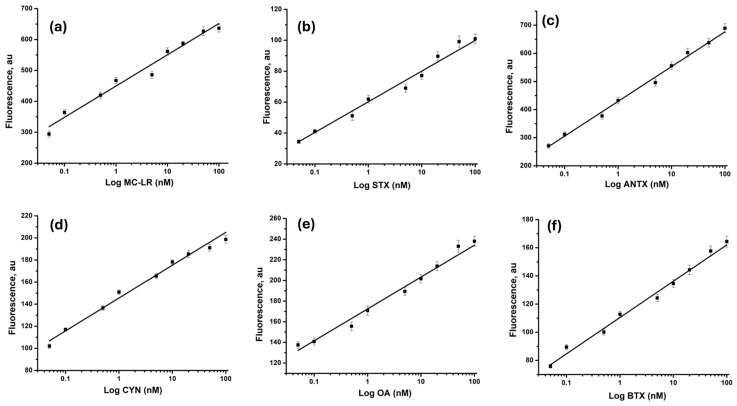
Calibration plots of the recorded fluorescence intensities as a function of the logarithm of cyanotoxins’ concentrations (nM): microcystin-LR (**a**), anatoxin (**b**), saxitoxin (**c**), cylindrospermopsin (**d**), okadaic acid (**e**), and brevetoxin (**f**).

The fluorescence spectra obtained in Figure 3 were used to plot the calibration curves for each toxin. For this, the fluorescence intensity (*I*) was plotted as a function of the logarithm of toxin concentrations. Figure 4a–f show the calibration curves for microcystin-LR, anatoxin-α, saxitoxin, cylindrospermopsin, okadaic acid, and brevetoxin, respectively.

The calibration curves show good linearity, with an excellent coefficient of determination (R^2^) and low detection limits (LODs), for the different toxins (Table 1). The fluorescent aptameric assay was applicable in the range of 0.05 to 100 nM for the six analytes.

Based on the regression equations, the limit of detection (LOD) was calculated for each toxin as 3σ/slope, where σ is the standard deviation of the blank samples. The obtained LODs, shown in Table 1, are much lower than those obtained in our previous work reporting the electrochemical detection of the same cyanotoxins [35].

### 2.3. Selectivity and Real Sample Applicability

**Figure 5:**
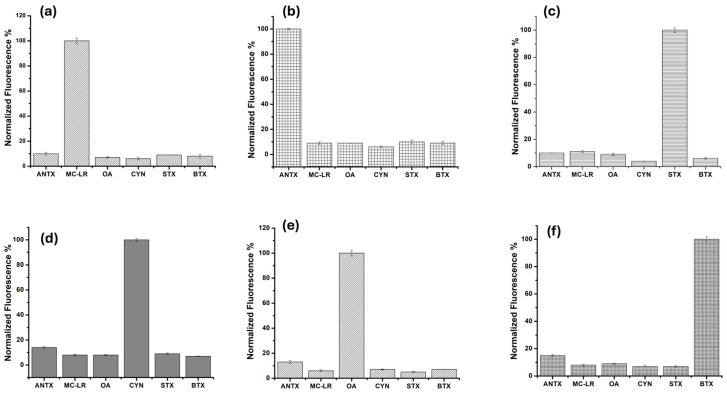
Cross-reactivity study of the aptameric assays for MC-LR (**a**), ANTX (**b**), STX (**c**), CYN (**d**) OA (**e**), and BTX (**f**) aptasensors towards the non-specific cyanotoxins (concentration of 5 nM).

Aiming to confirm the specificity of each aptameric assay for its targeted toxin, a cross-reactivity study was carried out. In brief, each duplex composed of FAM–aptamer/BHQ1-cDNA was incubated with the same concentration (5 nM) of its specific target and the five non-specific toxins, separately. The fluorescence intensity of the duplex was measured at 518 nm, before and after incubation with each toxin. Then, the percentage of fluorescence recovery was calculated as F = *I*/*I*_0_%, where I_0_ and I are the fluorescence intensities of the free duplex and the complex aptamer–cDNA–toxin, shown in Figure 5a–f. We observed a negligible fluorescence recovery of the duplexes for the non-specific toxins, in contrast to a significant recovery with the specific analyte. Therefore, we confirmed the absence of cross-reactivity of the different aptameric duplexes with the non-specific toxins.

Finally, we tested the application of our methodology in the detection of different toxins in real water samples. As shown in Table 2, excellent recoveries were obtained, with RSDs (relative standard deviations) lower than 5%.

## 3. Conclusions

Cyanotoxin testing is of great importance for ensuring water and food safety. Aptamer-based assays constitute an excellent alternative to the traditional techniques used for toxin detection, including chromatography and immunoassays. In this work, a fluorescent aptameric assay based on a duplex-to-complex structure-switching approach was successfully applied to quantify six marine toxins: microcystin-LR, anatoxin-α, saxitoxin, cylindrospermopsin, okadaic acid, and brevetoxin. We first investigated the quenching effect of the BHQ1-labeled cDNA sequences on the FAM-labeled aptamers, followed by fluorescence recovery analysis after binding of the six aptamers to their specific toxins. Under the optimized conditions, the homogeneous assay showed a wide linear range of quantification from 0.05 to 100 nM. In addition, high sensitivity was achieved, with LODs ranging from 0.026 to 0.15 nM. Moreover, no significant cross-reactivity was observed for the six aptameric assays, showing the high selectivity of the aptamers. Finally, we demonstrated the applicability of our method in water samples, achieving good agreement with the calibration curve established in buffer. Our approach constitutes a very good alternative for immunoassays and aptamer-based assays targeting a single analyte, particularly for toxins commonly present in the same environment.

## 4. Experimental

### 4.1. Chemicals and Instrumentation

The chemicals and instrumentation used in this work are described in Supplementary Materials.

### 4.2. Design of the Aptamer–cDNA Duplex

First, the duplex DNA was prepared by binding each aptamer sequence to its complementary DNA. For this, 350 µL of the FAM-labeled aptamer was incubated with the same volume of the BHQ1-labeled cDNA. A concentration of 500 nM of each sequence was used. The mixture was kept in a 90 °C water bath, followed by slow cooling to room temperature for 3 h in dark. This process decreases the distance between the fluorophore–quencher pair, thus enhancing the quenching of fluorescence. In parallel, a control mixture was prepared using the same protocol by incubating the FAM-labeled aptamers with binding buffer. Finally, the prepared duplexes were stored at 4 °C until further use.

### 4.3. Aptamer-Based Assay

Each duplex of the hybridized aptamer–cDNA pairs was used in the detection of its specific toxin. For this, the aptamer–cDNA (100 nM) was incubated with different concentrations of the targeted analyte ranging from 0.05 to 100 nM for 30 min. The fluorescence measurements were conducted before and after incubation with the increasing concentrations of the six toxins. To study the specificity of the proposed aptamer assay, the different duplexes were cross-reacted with the non-specific toxins at a fixed concentration of 5 nM, separately. Then, the corresponding fluorescence intensities were compared to those obtained with the specific toxin. Finally, the fluorescent assay was applied for the detection of the six toxins in real samples. Water samples, collected in Riyadh city, were spiked with different concentrations (10, 25, and 100 nM) of microcystin-LR, anatoxin-α, saxitoxin, cylindrospermopsin, okadaic acid, and brevetoxin. Fluorescence determination was carried out under the same conditions described above.

## Figures and Tables

**Table 1:** Analytical performance of the fluorescent aptameric assay for MC-LR, ANTX, STX, CYN, OA, and BTX.

Toxin	Linear Regression	R^2^	LOD ng/mL
MC-LR	*I* = 449.43 + 101.09 Log MC-LR concentration (nM)	0.97	0.15
ANTX	*I* = 429.11 + 123.65 Log ANTX concentration (nM)	0.99	0.06
STX	*I* = 60.19 + 19.83 Log STX concentration (nM)	0.99	0.075
CYN	*I* = 145.56 + 29.76 Log CYN concentration (nM)	0.99	0.027
OA	*I* = 172.4 + 30.88 Log OA concentration (nM)	0.98	0.041
BTX	*I* = 110.44 + 25.75 Log BTX concentration (nM)	0.99	0.026

**Table 2:** Application of the aptamer-based assay in the determination of microcystin-LR, anatoxin-α, saxitoxin, cylindrospermopsin, brevetoxin, and okadaic acid in spiked water samples.

Cyanotoxin	Spiking Concentration (nM)	Measured Concentration (nM)	Recovery Percentage (%)	RSD (%)
MC-LR	10	9.87	98.7	4.25
	25	25.02	102	2.21
	100	99.87	99.87	1.8
CYN	10	10.08	108	4.65
	25	24.92	99.68	4.9
	100	99.73	99.73	1.5
ANTX	10	10.06	106	3.8
	25	24.89	99.56	4.56
	100	98.97	98.97	3.78
STX	10	9.48	94.8	2.23
	25	24.91	99.64	4.27
	100	99.78	99.78	1.99
BVTX	10	10.1	110	4.89
	25	24.87	99.48	3.32
	100	99.23	99.23	4.81
OA	10	9.73	97.3	3.99
	25	25.08	108	1.76
	100	99.96	99.96	2.93

## Data Availability

The raw data supporting the conclusions of this article will be made available by the authors on request.

## Supplementary Materials

The following supporting information can be downloaded at: https://www.mdpi.com/article/10.3390/toxins16110476/s1, Table S1. Aptamers and cDNAs used in the fluorescent assay.
