# Supplementary material for: Design of a Duplex-to-Complex Structure-Switching Approach for the Homogeneous Determination of Marine Biotoxins in Water
Source: Toxins (Basel). 2024 Nov 4;16(11):476. doi: 10.3390/toxins16110476 (PMC11598080; doi:10.3390/toxins16110476)
Supplement: Supplementary file 1 [file toxins-16-00476-s001.zip › toxins-3255969-supplementary.pdf]

Supporting information:

Chemicals and instrumentation:

HPLC purified aptamers and complementary DNA sequences were provided by Metabion International (Planegg, Germany). The aptamers labelled with 6′ carboxyfluorescein and cDNA conjugated to the quencher black hole quencher (BHQ1) are presented in Table S.1. The DNA oligonucleotides were dissolved in ultrapure Milli-Q water to make the stock solutions and stored at -20 °C until further use. The DNA solutions used in the experiments were diluted with binding buffer. Microcystin-LR, anatoxin-α, saxitoxin, cylindrospermopsin, okadaic acid and brevetoxin sodium salt were purchased from Enzo Life Sciences (Ontario, Canada). The aptamers and toxins were diluted with binding buffer (50 mM Tris, pH 7.5, 150 mM NaCl, 2 mM $MgCl_2$).

The fluorescence measurements of the FAM-labelled aptamers were measured using Nanodrop ND3300 fluorospectrometer (Thermo Scientific, Canada) at an excitation wavelength of 480 ± 10 nm and an emission wavelength of 525 ± 10 nm. All the measurements were recorded in binding buffer at room temperature.

Table S.1. Aptamers and cDNAs used in the fluorescent assay.

Sequence name	Sequence and modification
Anti MC-LR aptamer	5'- GGC GCC AAA CAG GAC CAC CAT GAC AAT TAC CCA TAC CAC CTC ATT ATG CCC CAT CTC CGC-3' FAM
cDNA of MC -LR aptamer	5'- BHQ1 GCG GAG ATG G -3’
Anti OA aptamer	5'- GGT CAC CAA CAA CAG GGA GCG CTA CGC GAA GGG TCA ATG TGA CGT CAT GCG GAT GTG TGG-3' FAM
cDNA of OA aptamer	5'- BHQ1 CCA CAC ATC CG-3’
Anti CYN aptamer	5'- GGC ATC AGG CAA CAA CCG ATG GTC CGG CCA CCC TAA CAA CCA GCC CAC CCA CCA CCC CGC CG-3' FAM
cDNA of CYN aptamer	5'- BHQ1 CGG CGG GGT GG -3’
Anti ANTX aptamer	5'- TGG CGA CAA GAA GAC GTA CAA ACA CGC ACC AGG CCG GAG TGG AGT ATT CTG AGG TCG G-3' FAM
cDNA of ANTX aptamer	5'- BHQ1 CCG ACC TCA GAA-3’
Anti BTX aptamer	5'- GGC CAC CAA ACC ACA CCG TCG CAA CCG CGA GAA CCG AAG TAG TGA TCA TGT CCC TGC GTG-3' FAM
cDNA of BTX aptamer	5'- BHQ1 CAC GCA GGG ACA-3’
Anti SXT aptamer	5'- TTG AGG GTC GCA TCC CGT GGA AAC AGG TTC ATT G-3' FAM
cDNA of SXT aptamer	5'- BHQ1 CAA TGA ACC TGT T-3’
